# Supplementary material for: Interaction between Enrofloxacin and Three Essential Oils (Cinnamon Bark, Clove Bud and Lavender Flower)—A Study on Multidrug-Resistant Escherichia coli Strains Isolated from 1-Day-Old Broiler Chickens
Source: Int J Mol Sci. 2024 May 10;25(10):5220. doi: 10.3390/ijms25105220 (PMC11121375; doi:10.3390/ijms25105220)
Supplement: Supplementary file 1 [file ijms-25-05220-s001.zip › Supplementary Files Table S1.pdf]

Table S1. Drug susceptibility of selected isolates of *Escherichia coli* and the ATCC 25922 reference strain (source: official test reports; disk-diffusion method)

N°	*Escherichia coli*	antimicrobial agent								
		AMX	AMC	ENR	SXT	DO	OT	FFC	CT	LS
1.	MDR-1	R	R	R	R	R	R	R	S	R
2.	MDR-2	R	S	R	R	R	R	R	S	R
3.	MDR-3	R	R	R	R	R	R	R	S	R
4.	MDR-4	R	R	R	R	R	R	S	S	R
5.	MDR-5	R	R	R	R	R	R	S	S	R
6.	MDR-6	R	S	R	R	R	R	R	S	S
7.	MDR-7	R	S	R	R	R	R	S	S	R
8.	MDR-8	R	S	R	R	R	R	S	S	S
9.	MDR-9	R	S	R	R	R	R	S	S	S
10.	MDR-10	R	S	R	R	R	R	S	S	S
11.	SDR-1	R	S	I	I	R	R	S	S	S
12.	SDR-2	R	S	I	S	I	R	S	S	S
13.	SDR-3	R	S	I	R	S	S	S	S	S
14.	SENS-1	S	S	S	S	S	S	S	S	S
15.	SENS-2	S	S	S	S	S	S	S	S	S
16.	SENS-3	S	S	S	S	S	S	S	S	S
17.	ATCC 25922	S	S	S	S	S	S	S	S	S

R resistant   I intermediate   S susceptible

AMX – amoxicillin
AMC – amoxicillin/clavulanic acid
ENR – enrofloxacin
SXT – sulfamethoxazole/trimethoprim
DO – doxycycline
OT – oxytetracycline
FFC – florfenicol
CT – colistin
LS – lincomycin/spectinomycin
