# Supplementary material for: Interaction between Enrofloxacin and Three Essential Oils (Cinnamon Bark, Clove Bud and Lavender Flower)—A Study on Multidrug-Resistant Escherichia coli Strains Isolated from 1-Day-Old Broiler Chickens
Source: Int J Mol Sci. 2024 May 10;25(10):5220. doi: 10.3390/ijms25105220 (PMC11121375; doi:10.3390/ijms25105220)
Supplement: Supplementary file 1 [file ijms-25-05220-s001.zip › Supplementary Files Figure S3 and S4.pdf]

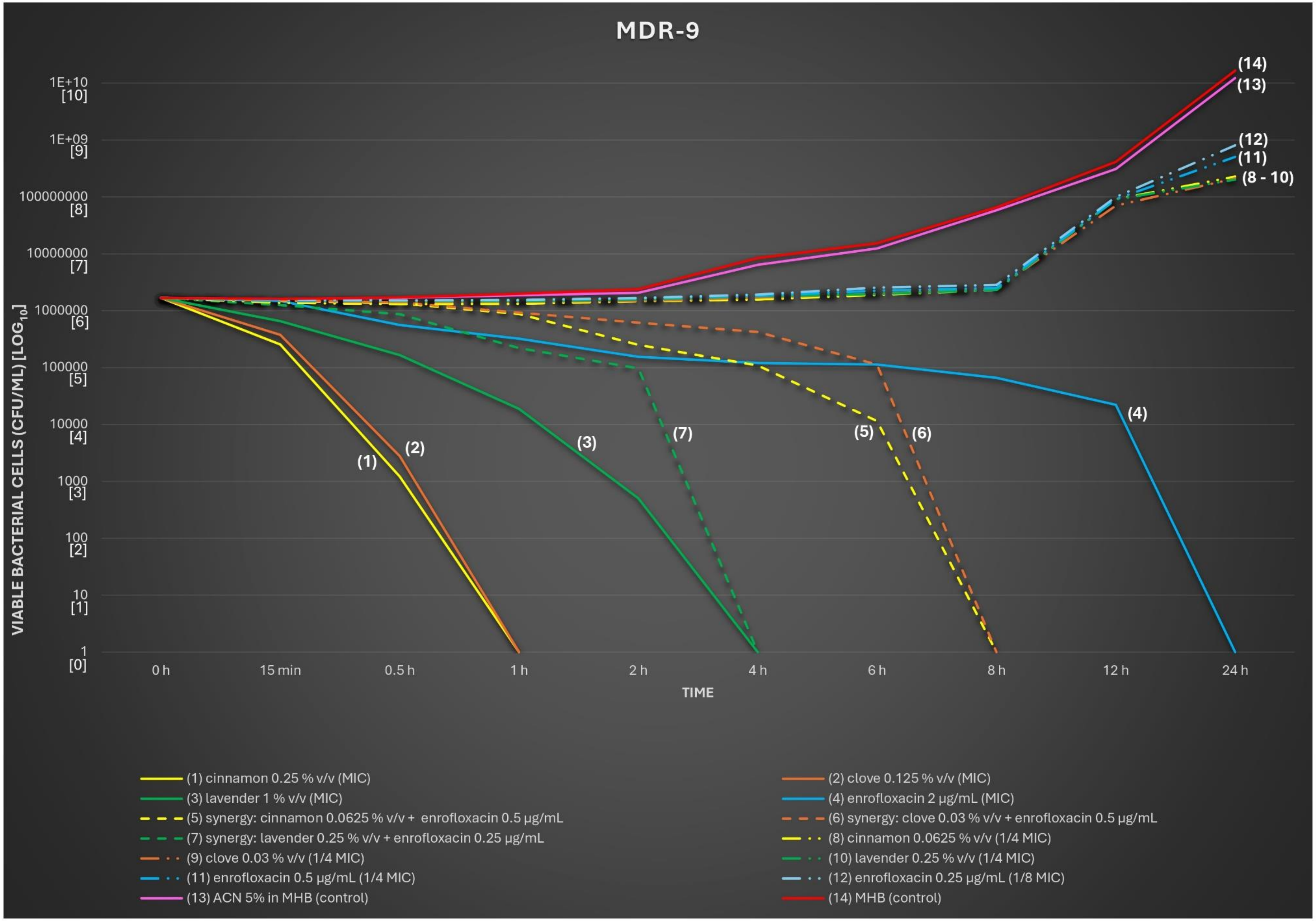

Figure S3 Time-kill analysis of cinnamon bark, clove bud and lavender flower essential oils administered alone and in the synergistic combination with enrofloxacin (*Escherichia coli* MDR-9 strain)

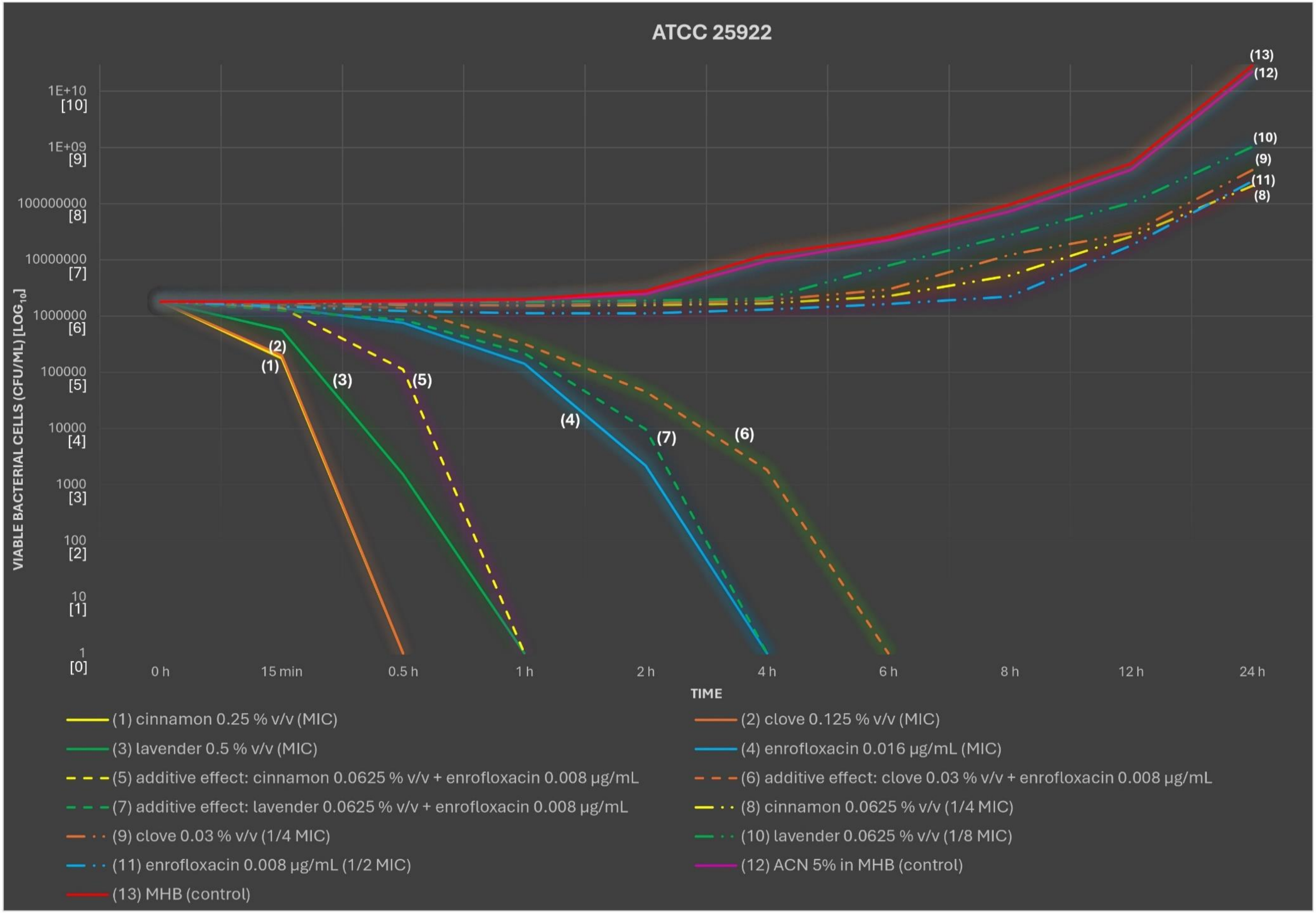

Figure S4 Time-kill analysis of cinnamon bark, clove bud and lavender flower essential oils administered alone and in combination with enrofloxacin (additive effect; *Escherichia coli* ATCC 25922 reference strain)
