# Supplementary material for: Interaction between Enrofloxacin and Three Essential Oils (Cinnamon Bark, Clove Bud and Lavender Flower)—A Study on Multidrug-Resistant Escherichia coli Strains Isolated from 1-Day-Old Broiler Chickens
Source: Int J Mol Sci. 2024 May 10;25(10):5220. doi: 10.3390/ijms25105220 (PMC11121375; doi:10.3390/ijms25105220)
Supplement: Supplementary file 1 [file ijms-25-05220-s001.zip › Supplementary Files Figure S1 and S2.pdf]

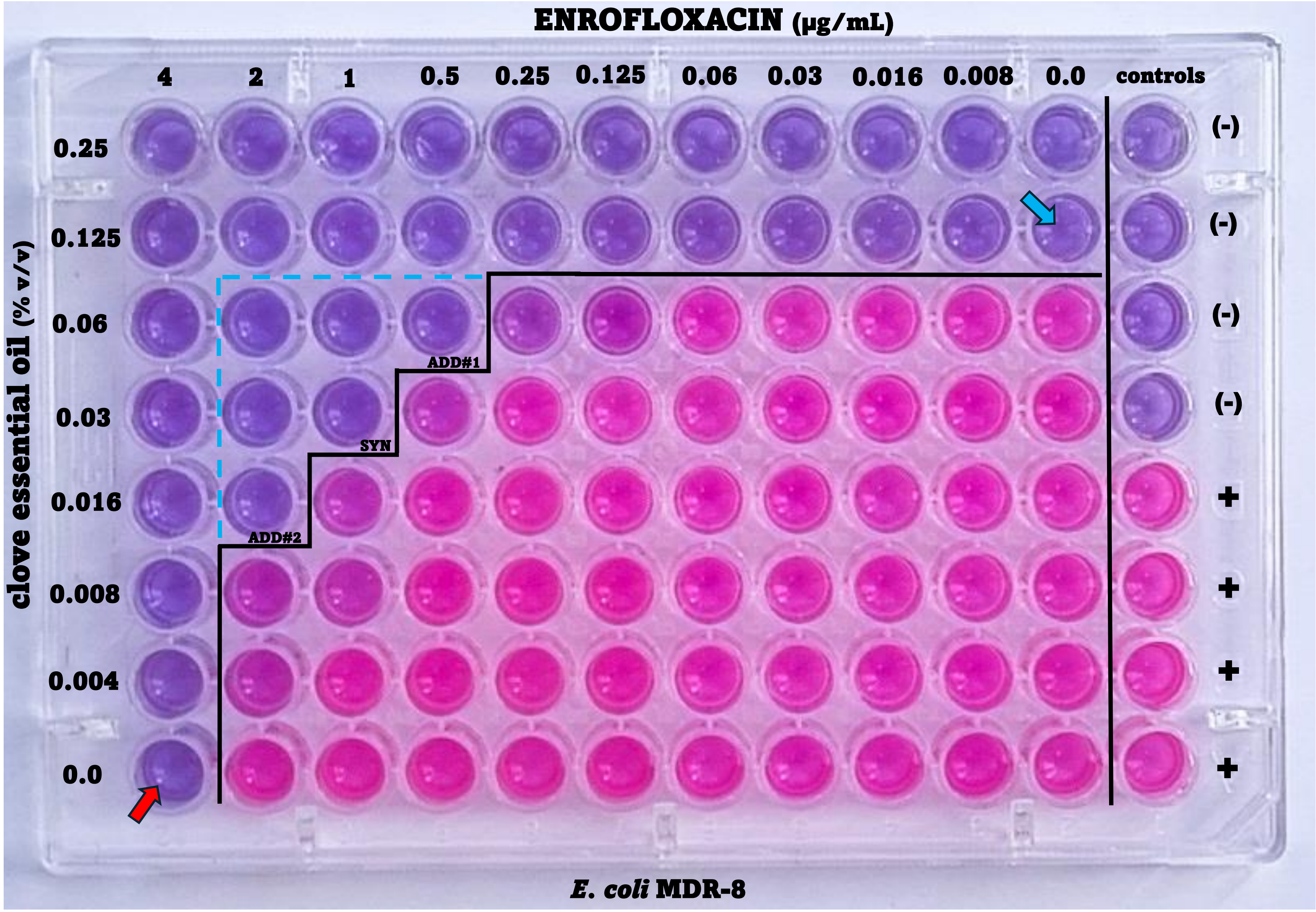

Figure S1 An example of stair-step "weak" synergism after 24 incubation (isolate MDR-8; clove EO)

Description: the light blue dashed lines mark the area where the interaction between enrofloxacin and essential oil occur (a total of six wells). Blue wells indicate 99.9% reduction of *E. coli*. Faint pink border wells indicate incomplete interaction (some percentage of *E. coli* survived the 18 h incubation). Intense pink wells indicate an ineffective antimicrobial agent (presence of live cells). ➡ individual MIC for clove essential oil (0.125 % v/v) ➡ individual MIC for enrofloxacin (4 µg/mL) (-) negative control + positive control
SYN - "weak" synergy (FICi =0.5) with an equal reduction of the effective dose
ADD#1 - additive effect (FICi = 0.625) to the maximal reduction of enrofloxacin concentrations
ADD#2 - additive effect (FICi = 0.625) to the maximal reduction of clove EO content

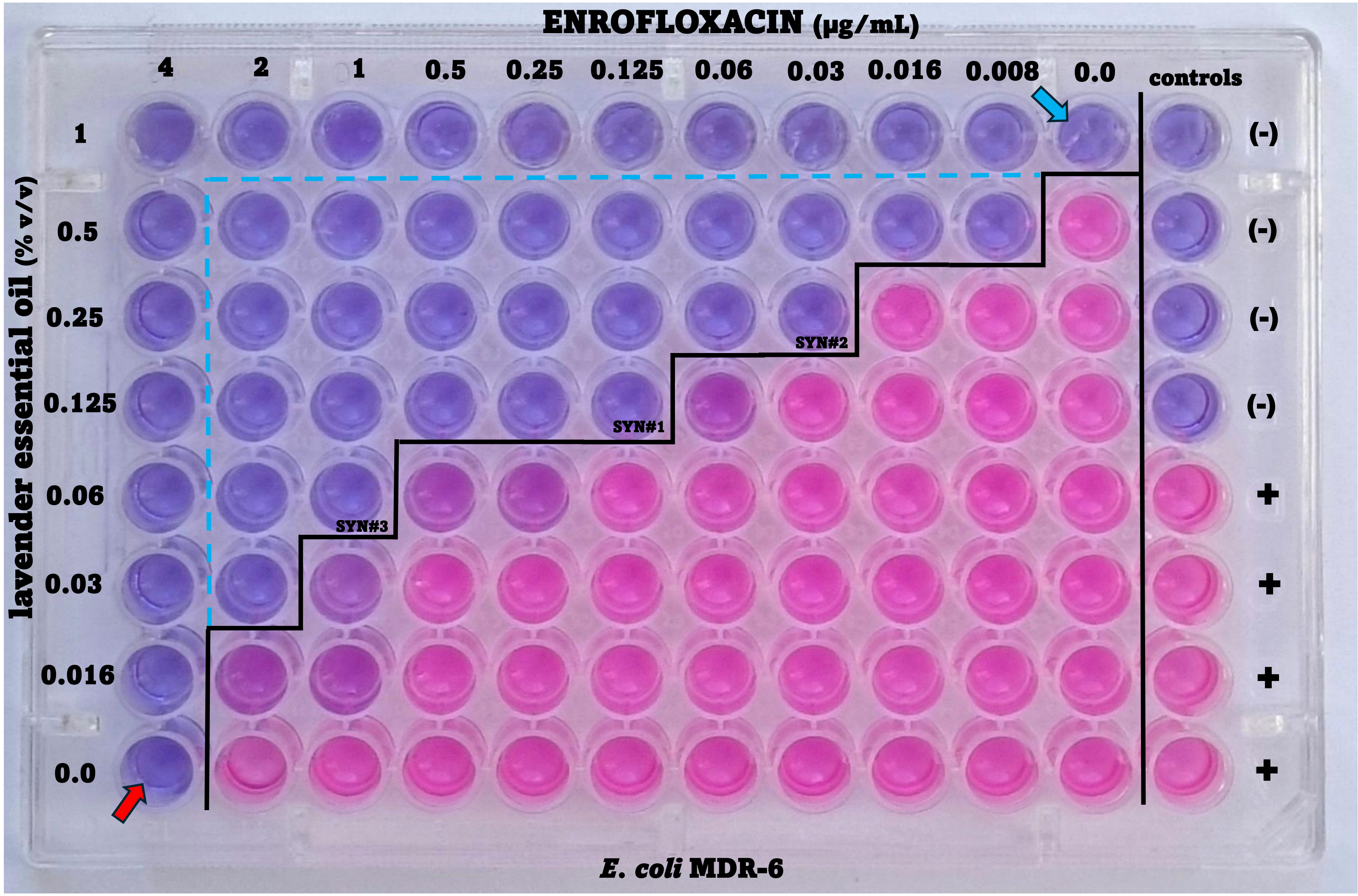

Figure S2 An example of "strong" synergism after 24 incubation (isolate MDR-6; lavender EO)

Description: the light blue dashed lines mark the area where the interaction between enrofloxacin and essential oil occur (a total of twenty-four wells). Blue wells indicate 99.9% reduction of *E. coli*. Faint pink border wells indicate incomplete interaction (some percentage of *E. coli* survived the 18 h incubation). Intense pink wells indicate an ineffective antimicrobial agent (presence of live cells). ➡ individual MIC for lavender essential oil (1 % v/v) ➡ individual MIC for enrofloxacin (4 µg/mL) (-) negative control + positive control

SYN#1 - "strong" synergy (FICi =0.155) with a reduction in lavender EO content to the level of the best results for clove EO and the isolate is becoming susceptible to enrofloxacin

SYN#2 - "medium" synergy (FICi = 0.258) to the maximal reduction of enrofloxacin concentrations with a reduction in lavender EO content to the level of the best results for cinnamon EO

SYN#3 - "medium" synergy (FICi = 0.31) to the maximal reduction of lavender EO concentration; however, the isolate becomes only weak intermediate to enrofloxacin
